# Comprehensive Gait Analysis and Kinetic Intervention for Overweight and Obese Children

**DOI:** 10.3390/children12020122

**Published:** 2025-01-23

**Authors:** Cristina Popescu, Daniela Matei, Anca Maria Amzolini, Magdalena Rodica Trăistaru

**Affiliations:** 1 Doctoral School, University of Medicine and Pharmacy of Craiova, 200349 Craiova, Romania; popescu.crist@yahoo.com; 2 Department of Medical Rehabilitation, University of Medicine and Pharmacy of Craiova, 200349 Craiova, Romania; rodica.traistaru@umfcv.ro; 3 Department of Medical Semiology, University of Medicine and Pharmacy Craiova, 200349 Craiova, Romania; anca.amzolini@umfcv.ro

**Keywords:** children obesity, gait parameters, pelvic kinematics, kinetic program

## Abstract

Background/Objectives: Childhood obesity is a critical public health issue associated with biomechanical and functional impairments that influence gait and physical performance. This study aimed to evaluate the impact of a six-month personalized kinetic program on gait parameters and pelvic kinematics in overweight and obese children. Methods: The prospective observational study included 100 children aged 8 to 15, divided into a study group (SG, n = 50) and a control group (CG, n = 50). The SG participated in a tailored kinetic program focusing on flexibility, strength, and coordination exercises, while the CG maintained their usual activities. The program consisted of 60 min sessions conducted three times per week over a six-month period. Gait parameters and pelvic symmetry indices were assessed using the BTS G-WALK system. Ethical approval was granted by the Ethics Committee of the University of Medicine and Pharmacy, Craiova, under approval no. 38/1 March 2022. Results: Significant improvements were observed in the SG, with increases in cadence (steps/min), walking speed (m/s), and pelvic symmetry indices across all planes (sagittal, frontal, and transverse) (*p* < 0.0001). In contrast, no significant changes were observed in pelvic symmetry indices in the CG (*p* > 0.01). The Spearman correlation matrix and heatmaps highlighted a strong correlation between improved gait parameters and participation in the kinetic program (correlation coefficient over 0.45). Conclusions: The findings demonstrate that a targeted kinetic program can significantly improve gait mechanics and pelvic kinematics in overweight and obese children. These results emphasize the importance of personalized exercise interventions in managing obesity-related gait abnormalities and improving functional mobility.

## 1. Introduction

The prevalence of pediatric obesity has risen to alarming levels in the 21st century, posing a global public health challenge. By 2022, over 390 million children and adolescents aged 5–19 years were overweight, of which 160 million were classified as obese [1]. Childhood obesity predisposes individuals to severe systemic complications, such as type 2 diabetes and cardiovascular diseases [2,3,4,5], as well as musculoskeletal issues like reduced mobility and joint stress [6].

One critical area of concern is how obesity alters the biomechanics of walking in children, particularly through increased joint load and altered gait patterns. The excess body weight intensifies stress on weight-bearing joints, such as the hips, knees, and ankles, potentially accelerating joint wear and leading to discomfort or skeletal malalignments like knee valgus angles and increased step width [7]. Pain from these conditions discourages physical activity, perpetuating a cycle of inactivity and weight gain [8].

Altered gait patterns also emerge in obese children as a compensatory mechanism for excess weight. Common adaptations include a wider stance, shorter stride length, increased knee and ankle flexion, and slower cadence. These changes destabilize walking mechanics, leading to inefficiency, higher energy expenditure, and faster fatigue. Over time, these adaptations can cause abnormal joint and muscle wear, increasing the risk of musculoskeletal disorders. Gait analysis, which provides detailed insights into walking mechanics, is an essential tool for understanding and addressing these challenges.

Key Aspects of Gait Analysis in Obese Children:

- Spatiotemporal Parameters: Obese children generally exhibit shorter stride lengths, slower walking speeds, and reduced cadence compared to their non-obese peers. Their walking is characterized by a longer stance phase and gait cycle, reflecting inefficient mobility. While the literature on these parameters is inconsistent, studies have consistently noted prolonged cycle durations and lower cadence in obese children [9,10,11]. For example, Porta et al. highlighted increased stance phase duration and step width due to excess weight [12]. Understanding these patterns is vital for designing effective interventions.
- Joint Load and Kinematics: Excess body weight places additional stress on weight-bearing joints, altering their biomechanics. McMillan et al. reported reduced hip and knee flexion during walking, along with pronounced valgus knee alignment [13]. Obese children often exhibit a flatter foot pattern during heel contact [14], increased hip flexion, and a wider range of ankle motion during the gait cycle [15]. These adaptations can lead to joint damage and pain, further limiting mobility.
- Energy Expenditure: Walking in obese children requires greater energy due to increased body mass and inefficient gait mechanics. Shultz et al. noted that obese children generate more energy through their hips, knees, and ankles to sustain normal walking patterns, contributing to quicker fatigue and lower physical activity levels [16].
- Balance and Stability: Obesity affects balance and stability, which are critical for effective gait. A shift in the center of gravity due to fat distribution, particularly around the trunk, can increase instability during walking. Song-hua et al. found that obese children exhibit reduced walking stability compared to their averageweight peers [17,18], underscoring the need for targeted interventions to improve postural control.
- Motor Skill Development: Obesity impairs motor skill development, including those necessary for proper gait mechanics, with long-term implications for physical health and activity. Clark et al. observed a strong correlation between obesity and reduced movement quality in children, emphasizing the need for early intervention to prevent permanent deficits [19,20].

Various tools and techniques are employed in gait analysis to understand these biomechanical changes: video analysis provides initial qualitative insights into gait abnormalities [21]; pressure mats and force plates measure forces exerted during walking, offering quantitative data on balance and load distribution [22]; 3D motion capture systems create detailed three-dimensional models of gait, widely used in research and clinical settings [8]; wearable sensors like accelerometers and gyroscopes provide real-time data on gait parameters, enabling continuous monitoring [12,23].

The combination of increased joint load, altered gait patterns, and compromised motor skills makes everyday activities such as walking, running, or climbing stairs challenging for obese children. This struggle often leads to a sedentary lifestyle, further diminishing quality of life and exacerbating physical and emotional health issues. Targeted interventions are critical to breaking this cycle.

Targeted kinetic programs focusing on weight management, gait correction, and muscle strengthening have shown promise in addressing these challenges. As an example, strengthening the knee extensors and hip abductors, combined with dynamic alignment exercises, can alleviate joint stress and improve gait mechanics [24]. Treadmill exercises with reduced weight-bearing conditions provide an opportunity to correct gait patterns without putting excessive stress on the joints, encouraging proper movement mechanics [25]. Integrative neuromuscular training programs improve gait biomechanics by focusing on strength, balance, and coordination [26].

Structured physical activity programs, supported by interdisciplinary approaches, demonstrate significant potential in addressing the biomechanical and functional challenges faced by obese children. Drawing from research on kinesitherapy and non-pharmacological treatments [27,28,29,30,31], such interventions not only alleviate pain and improve mobility but also promote overall well-being and an active lifestyle. These findings emphasize the importance of integrating targeted kinetic therapies into healthcare strategies to enhance quality of life and long-term health outcomes for this vulnerable population. However, there is limited research on the biomechanical impact of such programs, particularly their effects on spatiotemporal gait parameters and pelvis kinematics.

This study aims to fill this gap by analyzing the evolution of spatiotemporal gait parameters in obese children and the impact of controlled physical activity on the symmetry of pelvis kinematics during walking. Using the G-SENSOR inertial system, G-Studio software, and standardized movement protocols, this research is the first to examine the relationship between kinetic interventions and the symmetry index in pelvis movements. Findings from this work could guide the integration of targeted interventions into healthcare systems, emphasizing their potential benefits for improving mobility and reducing obesity-related musculoskeletal issues [32,33].

## 2. Materials and Methods

### 2.1. Design Overview

This prospective interventional study with a control group was conducted from September 2022 to June 2023 at the Pediatrics and Physical Medicine and Rehabilitation Departments of Filantropia Clinical Hospital, Craiova. The study included 100 overweight and obese children, ages 8–15, with no additional pathological conditions. At baseline (T1), each participant underwent a comprehensive evaluation comprising clinical, paraclinical, and functional assessments conducted by a multidisciplinary team.

Participants were divided into two groups: the Study Group (SG): 50 children who agreed to participate in a kinesiotherapy (KT) program, and the Control Group (CG): 50 children who continued their usual daily routines without the additional customized KT program.

The children in the SG engaged in a personalized KT program tailored to their age, compliance level, and physical abilities. This program was seamlessly integrated into their daily routines and sustained over six months. Conversely, the CG participants maintained their usual daily activities without any supplementary physical activity.

All participants and their parents received educational guidance on obesity management, collectively referred to as educational measures (EM) and general primary preventive physical activity (GPPPA). These measures were designed to encourage healthier lifestyles and included the following: avoiding breakfast skipping [34], adopting healthy eating habits, including the “traffic light” diet [33], following the 5-2-1-0 Rule (5 servings of fruits and vegetables daily, no more than 2 h of screen time daily, at least 1 h of physical activity daily, and 0 sugary drinks daily [35]), engaging in screen-based physical activities (e.g., exergames) or other general preventive physical activities [36,37], ensuring adequate sleep according to age-specific recommendations [38]. Participants or parents reported their adherence to these strategies, providing insights into compliance with the educational measures.

**Figure 1:**
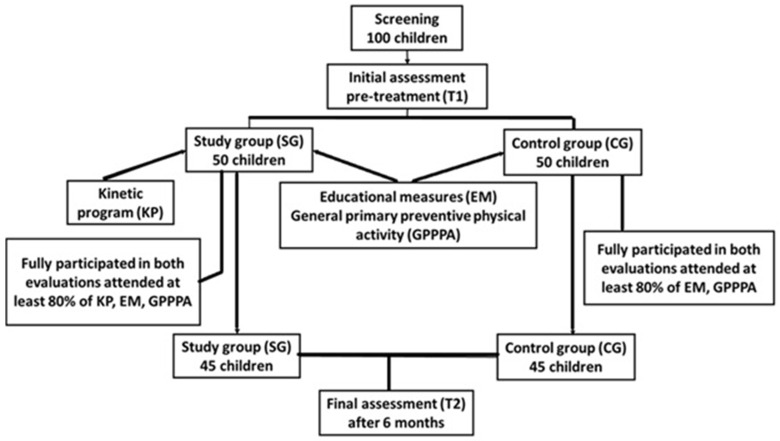
Diagram of study.

A follow-up assessment (T2) was conducted six months after the initial evaluation to measure the effects of the intervention. For statistical validity, only participants who met the following criteria were included in the final analysis (Figure 1):

- Completed both baseline (T1) and follow-up (T2) evaluations;
- Attended at least 80% of the kinesiotherapy sessions (for SG participants) and at least 80% of EM and GPPPA (for SG and CG children);
- Provided valid and complete data during the study period.
- In total, 45 children (90%) from the SG successfully completed the intervention and follow-up evaluation, while 48 children (96%) from the CG attended the follow-up assessment.

The impact of the intervention was assessed using validated tools, with comparisons made between the SG and CG: anthropometric data, spatio-temporal parameters, general kinematic parameters, symmetry index, propulsion index, and pelvis kinematics. Furthermore, the groups were examined based on age, gender, and background to confirm consistency and maintain the internal validity of the results. The selected age range of 8–15 years encompasses critical developmental stages in children. School-age children and adolescents undergo significant physical, cognitive, and social development, making this a critical period to address obesity-related gait abnormalities and promote lifelong healthy habits. This age group also captures a broad spectrum of gait development, allowing the evaluation of the kinesiotherapy program’s impact across different maturity levels.

The sample size was determined to balance feasibility and statistical power. The formula for calculating the required sample size for the Wilcoxon signed-rank test is based on the expected effect size and the desired power of the test. A commonly used formula for estimating the sample size for non-parametric tests like the Wilcoxon signed-rank test is

$$N=\frac{Z\alpha /2+Z\beta }{d}$$

In this formula, N is the required sample size per group; Zα/2 = 1.96 (alpha = 0.05) is the z-score corresponding to the desired significance level (alpha); Zβ = 0.84 (for 80% power) is the z-score corresponding to the desired power (1 − beta); d = 0.843 is the expected effect size (the calculated average effect size of approximately 0.843 indicates a strong relationship between the specified parameters in the “Study Group”). The required sample size for the Wilcoxon signed-rank test, calculated using the provided values, is approximately 35 participants per group. To account for potential dropouts and variability in adherence, we increased the sample size to 50 participants per group (a total of 100 participants) to ensure sufficient power for detecting meaningful differences. The observed dropout rates (10% in the Study Group and 4% in the Control Group) were minimal and did not compromise the validity of our statistical analyses or conclusions.

### 2.2. Participants

Inclusion Criteria: Participants were eligible for the study if they met the following conditions: (a) Provided written informed consent from a parent or guardian and verbal assent from the child. (b) Aged between 8 and 15 years. (c) Body mass index (BMI) exceeding the 85th percentile for age and sex, as determined by standardized growth charts. (d) Assessed as clinically healthy by a pediatric specialist. (e) Able to understand and communicate in the national language. (f) No prior experience with kinesiotherapy. (g) No participation in sports activities outside of regular school activities. (h) Able to walk independently and respond appropriately to instructions.

Exclusion Criteria: Participants were excluded from the study if they met any of the following conditions: (a) Recent traumatic injuries. (b) Diagnosed orthopedic, respiratory, or neurological conditions that could impede participation in physical activities (applicable to the SG). (c) Lack of compliance or willingness to engage in the daily kinesiotherapy program (for SG participants). (d) Inability to understand or adhere to the dietary guidelines provided by the medical team. (e) Lack of compliance with the EM and/or GPPPA.

Eligible participants and their parents or guardians were thoroughly informed about the study objectives, procedures, and their roles in the research.

Prior to enrollment in the study, a comprehensive assessment was conducted for each participant. This evaluation included a detailed examination of gait patterns, focusing on abnormalities or deviations related to obesity; spinal alignment, with particular attention to the frequent accentuation of lumbar lordosis observed in this population; and lower limb joints, including the hips, knees, ankles, and feet, to identify any functional limitations or structural concerns.

These evaluations were performed in alignment with established clinical practice guidelines for managing obesity in children [33]. This rigorous assessment ensured that all participants met the study’s criteria and provided a baseline for subsequent analyses.

### 2.3. Study Intervention

The kinesiotherapy program for the SG was initiated at the Physical Medicine and Rehabilitation Department under the supervision of a specialized physiotherapist. To align with the Intensive Health Behavior and Lifestyle Treatment (IHBLT) framework [39], the kinesiotherapy program (the first 12 sessions) was closely monitored by the physiotherapist to ensure adherence, safety, and the correct execution of exercises. The program acknowledged that managing obesity in children requires a greater investment of time and resources than routine pediatric care. Comprehensive monitoring and individualized attention were critical for achieving sustained behavior changes and maximizing the effectiveness of the intervention. The program was designed to integrate seamlessly into the children’s daily lives, encouraging participation in simple and manageable physical activities without disrupting their regular routines.

**Figure 2:**
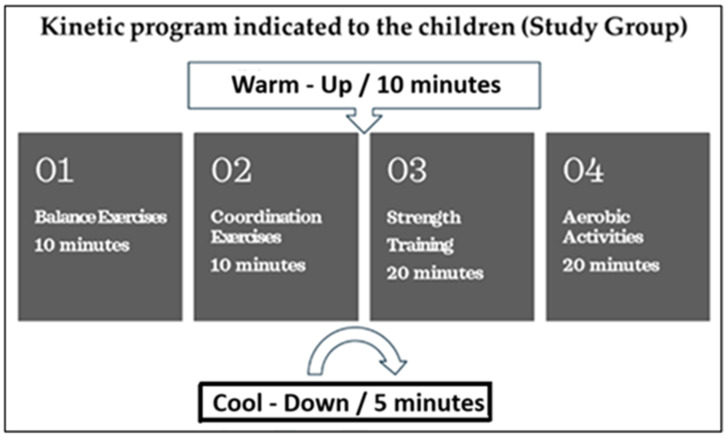
Kinesiotherapy program structure for the study group.

Program Design and Implementation: The kinesiotherapy program was patient-centered, based on each child’s activity behavior, assessed using the Compendium of Physical Activities developed by Ainsworth et al. [40]. The program consisted of 60 min sessions conducted three times per week over a six-month period. These sessions complemented the regular physical education classes included in the children’s school schedules. Figure 2 illustrates the structure of the kinesiotherapy program implemented for the Study Group, highlighting the key phases—warm-up, training activities (balance, coordination, strength, and aerobic exercises), and cool-down—with their respective durations.

The activities were specifically tailored to each child’s needs, abilities, and activity levels as determined by the physiotherapist in collaboration with the participants and their families. Physical activity intensity was carefully adjusted to ensure safety and optimal engagement.

The kinesiotherapy sessions focused on:

- Flexibility exercises: to improve joint mobility and reduce stiffness; these stretching routines are important to enhance muscle elasticity and prevent injuries;
- Adapted strength training: designed to suit the children’s age and physical abilities.

Weekly monitoring by the same physiotherapist ensured the correct execution of exercises and allowed for any necessary adjustments to the program. This structured and adaptable approach aimed to support physical development while encouraging long-term adherence to healthy physical activity habits (Table 1).

The intensity of these activities was monitored using smartwatches worn by each participant, which tracked their heart rates during the aerobic sessions. Target heart rate zones were calculated based on age-appropriate recommendations, ensuring that the activities fell within the moderate-intensity range (50–70% of maximum heart rate). We also referred to the Compendium of Physical Activities developed by Ainsworth et al., which provides standardized MET (metabolic equivalent of task) values for various activities. The selected activities (bike riding, brisk walking, and light jogging) were chosen based on their MET values corresponding to moderate-intensity aerobic exercise.

We took into account that resistive exercise is recommended for children over 16 years old [41].

### 2.4. Parameters and Measurements

The targeted assessment for the purpose of the present study was conducted initially (T1) at the beginning of the study and after six months (T2). The following parameters were taken into account—anthropometric data, spatio-temporal parameters, and pelvic kinematics were assessed for all subjects.

Anthropometric Data: attributes measured were height, weight, and BMI = weight (kg)/height^2^ (m). Body weight (kg) was measured using an electronic scale with a scale of 0 to 150 kg and an accuracy of 100 g. Standing height was measured using a portable stadiometer with an accuracy of 0.1 mm. During the measurement, the children were barefoot and with the least amount of clothing possible (only a light shirt and shorts). All anthropometric variables were assessed twice by a single examiner.

Gait analysis: conducted using the BTS G-WALK/BTS G-SENSOR 2 wireless system (BTS Bioengineering Corp., Garbagnate, Italy). This system utilizes an inertial sensor comprising a triaxial accelerometer, a magnetic sensor, and a triaxial gyroscope worn by the participant to enable functional gait analysis. The system’s specifications and procedures are outlined in the BTS G-SENSOR 2 hardware manual (version 1.2.2, Document Number: ERGS2-01271-06, 2016) and the BTS G-WALK^®^ user manual (version 9.0.0, Document Number: ERGSN-01134-20, 2018).

Protocol and Procedures: The Walk+ protocol was used for this study to ensure rapid and precise gait analysis. This protocol provides detailed insights into the following:

- Spatio-temporal parameters: overall and side-specific metrics for the right and left sides;
- General kinematic parameters: including symmetry and propulsion indices;
- Pelvic kinematics: analyzing pelvic motion across three planes (sagittal, frontal, and transverse).

To ensure reliable and repeatable data collection, the inertial sensor was positioned at the lumbosacral junction, specifically below the line connecting the two dimples of Venus, corresponding to the S1–S2 vertebrae.

Before data acquisition, children were given clear instructions on performing the test. They were asked to do the following:

- Stand still for a few seconds to allow proper calibration of the sensor;
- Walk at their natural speed along a straight path (>7 m) to ensure at least five complete gait cycles were recorded.

The software automatically detected and excluded any discontinuities in the gait pattern to enhance data accuracy.

For this study, the following gait parameters were analyzed:

- Global spatio-temporal parameters.
  1. Stride length (m);
  2. Cadence (steps/min);
  3. Average walking speed (m/s).
- Pelvic kinematics.
  1. Average angles of pelvic motion in the sagittal, frontal, and transverse planes;
  2. Symmetry of pelvic movement: symmetry index for pelvic tilt (SIT); symmetry index for pelvic rotation (SIR); symmetry index for pelvic obliquity (SIO).

**Figure 3:**
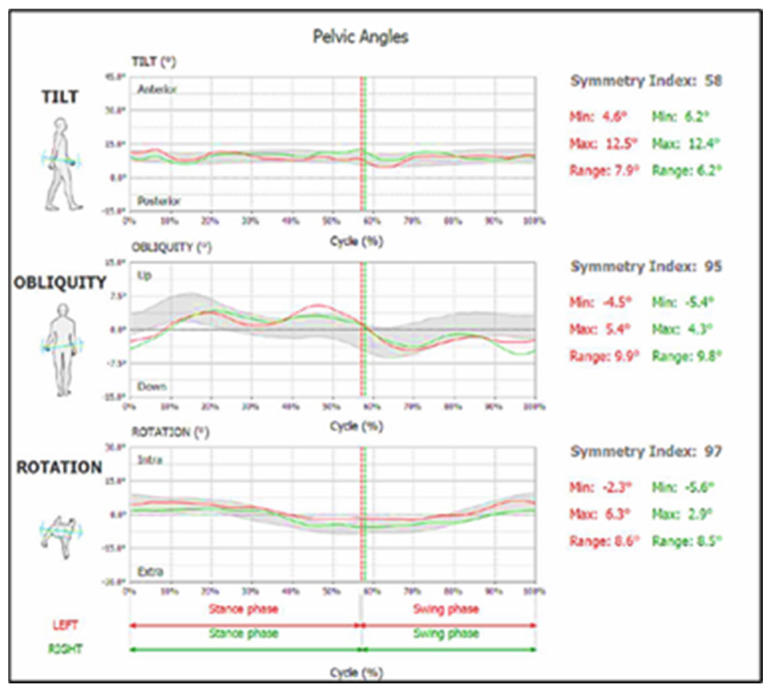
Pelvic angles and symmetry index established in the Walk Analysis Report (Walk+ protocol).

The symmetry indices used in this study evaluate the angular movement of the pelvis, focusing on left/right symmetry in each plane of motion (Figure 3). Unlike most gait analysis techniques, which primarily focus on the lower extremities (e.g., motion capture systems or electronic walkways), this approach provides a more comprehensive assessment by analyzing whole-body movement through the center of mass accelerations.

A control group of normal-weight children was not included in this study, as the evaluation device (BTS G-WALK/BTS G-SENSOR 2 wireless system, BTS Bioengineering Corp., Garbagnate Milanese, Italy) provides reference values through the Walk Analysis Report, making direct comparisons unnecessary.

### 2.5. Ethics Approval

This study placed the highest priority on ensuring the safety and well-being of all participants, particularly considering that children are a vulnerable population. Participants and their parents were fully informed about the study’s purpose, potential benefits, risks, and the measures taken to maintain confidentiality, in accordance with data protection regulations. They were also clearly informed of their right to withdraw from the study at any time, without providing a reason or facing any negative consequences.

Written informed consent was obtained from parents or guardians, and verbal assent was obtained from children after providing clear and age-appropriate explanations. The study strictly followed the principles outlined in the Declaration of Helsinki and Good Clinical Practice guidelines. Ethical approval was granted by the Ethics Committee of the University of Medicine and Pharmacy of Craiova, under approval no. 38/1 March 2022.

### 2.6. Statistical Analysis

Data collected during the study were organized in a Microsoft Excel spreadsheet. Statistical analysis was performed using Microsoft 365 Excel 2021 and SPSS version 26.0. Continuous variables were presented as means and standard deviations (SD), while categorical variables (e.g., gender and location) were expressed as percentages.

Normality Testing: The Shapiro–Wilk test was used to assess whether the data followed a normal distribution. Results showed that many parameters deviated significantly from normality, as indicated by *p*-values less than 0.05.

Statistical Methods:

- First Between-Group Comparisons: The Mann–Whitney U test, a non-parametric method, was used to compare initial and final values between the Study Group and Control Group. This test was selected due to the non-normal distribution of many parameters;
- Within-Group Comparisons: The Wilcoxon signed-rank test was applied to compare initial and final values within each group. This test is ideal for paired data and does not require the assumption of normality;
- Correlation Analysis: a Spearman correlation was used to explore relationships between variables.

Significance Threshold: A *p*-value of less than 0.05 was considered statistically significant for all tests.

## 3. Results

### 3.1. The Demographic and Anthropometric Data

The demographic characteristics of the children in the study are summarized in Table 2. The average age was comparable between the two groups: 10.4 years in the SG and 10.9 years in the CG, with no statistically significant difference (*p* = 0.1145). All participants in both groups were between 8 and 15 years old.

The urban-to-rural ratio was 23:22 (1.04) in the SG and 27:21 (1.28) in the CG, with no statistically significant differences (*p* = 0.2624 for urban participants and *p* = 0.3124 for rural participants).

Gender Distribution: In the SG, 22 boys (49%) and 23 girls (51%) were included, while the CG had 23 boys (48%) and 25 girls (52%). The girl-to-boy ratio was similar across the two groups, with no statistically significant differences (*p* = 0.2172 for girls and *p* = 0.2621 for boys).

BMI Classification: Participants classified as overweight had a BMI between the 85th and 95th percentiles, while those classified as having grade 1 obesity had a BMI ≥ 95th percentile.

- First SG: 11 girls and 12 boys were overweight, while 12 girls and 10 boys had grade 1 obesity;
- CG: 16 girls and 13 boys were overweight, while 9 girls and 10 boys had grade 1 obesity.

The *p*-value (*p* = 0.2736) indicated no statistically significant differences in BMI classification between the groups, confirming the homogeneity of the study population.

An analysis of BMI by gender and residence revealed no statistically significant differences (*p* > 0.1), indicating that the residential area did not influence BMI.

### 3.2. Study Group, Time-Evolution

**Figure 4:**
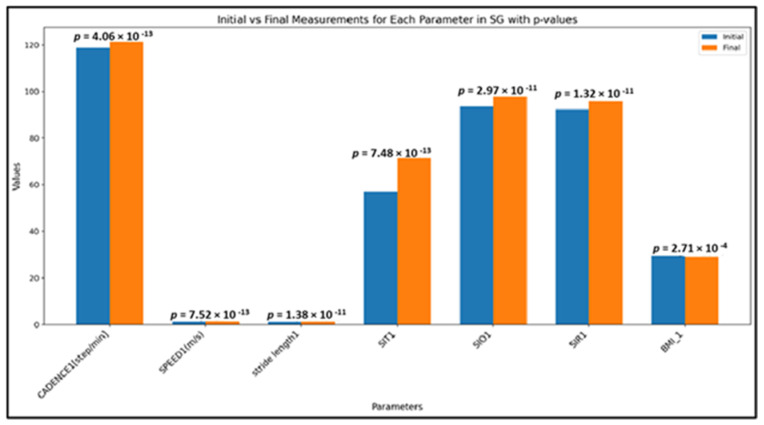
Mean values of all studied parameters in the SG, in both assessment moments (initial/final); SIT—symmetry index for pelvic tilt motion; SIO—symmetry index for pelvic obliquity motion; SIR—symmetry index for pelvic rotation motion; BMI = body mass index) (the Wilcoxon signed-rank test used for *p*-values).

The results indicate significant differences (we applied the Wilcoxon signed-rank test) between the initial and final measurements for all parameters tested (Figure 4).

Significant changes were observed across all the global spatio-temporal parameters studied:

- Cadence (steps/min): a highly significant improvement in cadence was noted (*p* < 0.0001), indicating that the intervention had a substantial effect on step frequency;
- Speed (m/s): walking speed also showed a significant increase (*p* < 0.0001), reflecting enhanced movement efficiency;
- Stride length (m): significant changes in stride length (*p* < 0.0001) were identified, suggesting modifications in gait mechanics over the intervention period.

Pelvic Kinematics: significant improvements were found in all symmetry indices (*p* < 0.0001), suggesting enhanced balance and coordination in gait mechanics as a result of the intervention.

BMI Changes: the Wilcoxon signed-rank test produced a statistic of 556.0 with a *p*-value of 0.00027, confirming a statistically significant reduction in BMI over the course of the study.

Comparison by Gender and Residence: No significant differences were observed in the studied parameters between urban and rural participants or between boys and girls. Independent *t*-tests showed that *p*-values for comparisons across these groups exceeded the standard threshold for statistical significance (*p* > 0.05), indicating uniform responses to the intervention regardless of gender or residential background.

### 3.3. Control Group, Time-Evolution

**Figure 5:**
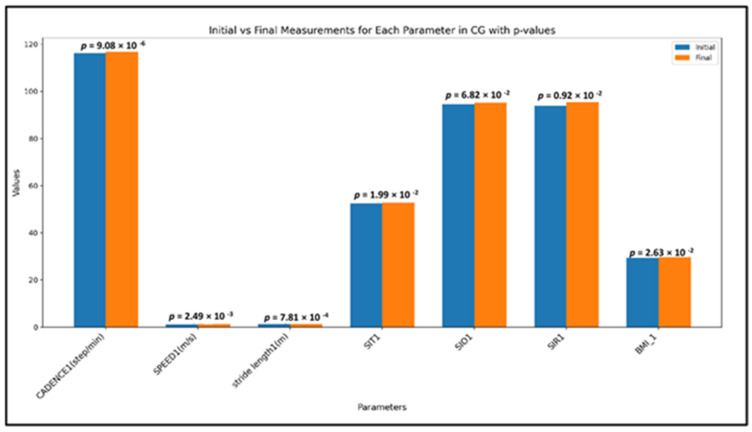
Mean values of all studied parameters in CG, in both assessment moments (initial/final); SIT—symmetry index for pelvic tilt motion; SIO—symmetry index for pelvic obliquity motion; SIR—symmetry index for pelvic rotation motion; BMI = body mass index; the Wilcoxon signed-rank test was used for *p*-values.

For the Control Group (CG), statistically significant differences were identified only in the global spatio-temporal parameters. However, the mean values showed minimal improvement compared to the substantial progress observed in the Study Group (SG) (Figure 5).

The specific changes in the global spatio-temporal parameters with statistical significance are as follows:

- Cadence (steps/min): a significant increase was observed (*p* = 9.08 × 10^−6^), indicating slight alterations in the participants’ step frequency;
- Speed (m/s): significant changes in walking speed were noted (*p* = 0.0025), suggesting minor improvements in movement efficiency, which may be attributed to natural adaptations or external factors during the six-month study period;
- Stride Length (m): changes in stride length were significant (*p* = 0.00078), reflecting modest adjustments in gait mechanics over time.

Pelvic Kinematics: no significant changes were observed in pelvic symmetry indices, highlighting limited progress in balance and coordination: SIT: *p* = 0.199, SIR: *p* = 0.092, SIO: *p* = 0.682.

BMI Changes: significant changes in BMI were recorded (*p* = 0.026), suggesting natural weight fluctuations within the CG over the study period.

### 3.4. Study Group Versus Control Group

The Mann–Whitney U test was applied to compare the initial values of the global spatio-temporal parameters (cadence, speed, stride length) and pelvic kinematics (symmetry indices) between the Study Group (SG) and the Control Group (CG) (Table 3).

The Pre-Treatment Evaluation (T1):

The T1 analysis revealed no statistically significant differences between the SG and the CG across the measured global spatio-temporal parameters:

- Cadence (steps/min): U-statistic = 2542, *p* = 0.135;
- Speed (m/s): U-statistic = 2422, *p* = 0.341;
- Stride Length (m): U-statistic = 2269.5, *p* = 0.790.
- Similarly, no significant differences were found for pelvic symmetry indices:
- SIT: *p* = 0.220;
- SIO: *p* = 0.685;
- SIR: *p* = 0.484.

For BMI, the comparison yielded a U-statistic of 2132 and a *p*-value of 0.727, indicating no significant difference between the groups.

These findings confirm that the initial conditions of both groups were statistically comparable across all parameters, ensuring that any observed changes during the study can be attributed to interventions or natural variations rather than baseline differences.

Final Evaluation (T2):

Regarding global spatio-temporal parameters, the following results were observed:

- Cadence (steps/min): U-statistic = 2751.0, *p* = 0.015. A statistically significant difference was observed, with the SG showing higher cadence compared to the CG, suggesting differences in walking patterns potentially influenced by the kinetic program;
- Speed (m/s): U-statistic = 2966.5, *p* = 0.0007. This significant difference indicates that the SG exhibited higher walking speeds, reflecting potential improvements in physical performance or capability;
- Stride Length (m): U-statistic = 2611.5, *p* = 0.071. The lack of statistical significance suggests that stride length variations between the groups were likely random.

Pelvic Symmetry Indices: Highly significant differences were observed in pelvic kinematics, pointing to variations in stability and rhythmic movement:

- First SIT: U-statistic = 3521.0, *p* = 3.62 × 10^−9^;
- Second SIO: U-statistic = 3823.0, *p* = 1.31 × 10^−13^;
- Third SIR: U-statistic = 3084.0, *p* = 5.08 × 10^−5^.

These findings suggest significant improvements in the oscillatory movement and regularity of pelvic motion during gait in the SG, potentially reflecting enhanced stability and coordination due to the intervention.

BMI comparison: The U-statistic for BMI was 1821.0, with a *p*-value of 0.077, indicating no statistically significant difference between the groups. This suggests that changes in BMI were likely due to random variation rather than systematic differences caused by the intervention.

**Figure 6:**
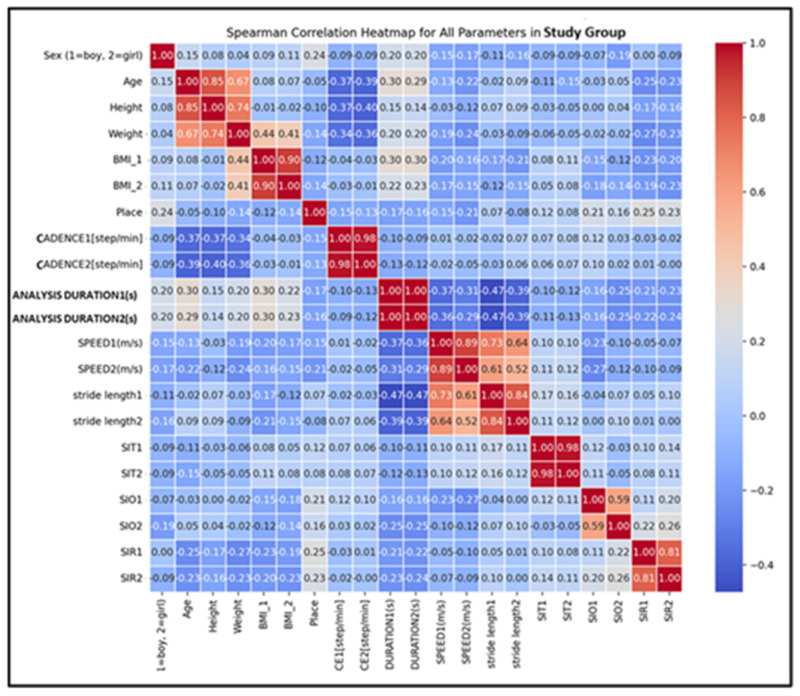
Correlation between studied parameters in the SG (the Spearman correlation test).

To support the efficiency of the applied kinetic program, it was also performed correlations between the spatio-temporal parameters and pelvic kinematics for the SG, both initially and at the end (Figure 6).

The Spearman correlation matrix and heatmaps revealed a few significant relationships between the studied parameters.

Correlation Between Speed and Stride Length:

- Initial Correlation Coefficient: 0.734;
- Final Correlation Coefficient: 0.521 bullet.

These strong positive correlations (absolute values > 0.5) indicate that as speed increases, stride length also tends to increase. This relationship is intuitive, as faster movements generally require longer strides. This finding is particularly relevant in gait analysis, as it highlights a fundamental biomechanical relationship between speed and stride length, reflecting core dynamics of movement efficiency.

Correlation Between SIT(T1) (Symmetry Index for Pelvic Tilt) and SIR(T1) (Symmetry Index for Pelvic Rotation): Correlation Coefficient: 0.097.

Although relatively weak, this positive correlation suggests a slight tendency for higher timing consistency -SIT(T1) to be associated with greater regularity in pelvic rotation -SIR(T1). While limited in strength, this relationship may still hold value in studies examining pelvic kinematics.

Correlation Between Stride Length (Initial and Final) and SIT (Initial and Final):

- Stride Length (T1) and Stride Length (T2): Correlation Coefficient = 0.84;
- SIT(T1) and SIT(T2): Correlation Coefficient = 0.98.

These strong positive correlations suggest a high degree of consistency in stride length and pelvic tilt symmetry over time. This consistency points to the effectiveness of the kinetic program in producing measurable and sustained improvements in these parameters.

No other significant correlations were identified within the dataset.

## 4. Discussion

Analyzing the walking patterns of overweight and obese children is essential because daily walking is often recommended to increase their physical activity and energy expenditure. Understanding the physiological and biomechanical factors behind their altered gait patterns can help determine whether walking is a safe and effective intervention for them. It also provides a scientific foundation for designing personalized rehabilitation strategies [42,43]. Research has shown that carrying excess body weight can have two significant impacts on overweight or obese children: biomechanical effects on gait: excess weight alters how these children walk, affecting their movement patterns [43]; impact on development: excess body weight can influence their musculoskeletal and neurosensory development, potentially leading to long-term functional and structural issues [44]. This understanding underscores the need for targeted interventions to address the unique challenges faced by this population.

The analysis of spatio-temporal gait parameters, such as cadence, walking speed, and stride length, revealed patterns consistent with findings from other studies focusing on overweight and obese children. Research has shown that children with higher body weight often adopt slower walking speeds and reduced cadence as adaptive strategies to minimize energy expenditure and mechanical strain during locomotion [45,46]. These findings align with those of Dufek et al. [47], who observed significant differences in cadence and speed between normal-weight and obese adolescents under various conditions. Similarly, Hung et al. [48] reported shorter normalized stride lengths in overweight children, particularly under dual-task conditions, further emphasizing the complexity of gait patterns in this population.

Meta-analyses on gait patterns in overweight and obese children consistently highlight the prolonged stance phase and increased step width as characteristic features of their gait [45,47,49]. These findings are supported by Pathare et al. [50] and Cimolin et al. [51], who observed prolonged load response phases and increased total support duration in obese children. However, findings on cadence and stride length remain inconsistent, likely due to variability in study designs and populations. While some studies, such as those by Hung et al. [48], emphasize shorter step lengths under dual-task conditions, others, like Dufek et al. [47], highlight the role of self-selected walking speeds in revealing unique gait adaptations.

Stride length, which showed limited improvement in the current study, has also been inconsistently reported in the literature. Pathare et al. [50] and Cimolin et al. [51] found no significant differences in stride length when comparing normal-weight and obese children, while studies like those by Dufek et al. [47] and Hung et al. [48] observed notable differences when varying walking speeds or task conditions. These inconsistencies suggest the need for further research into factors influencing stride length in overweight and obese populations.

Pelvic kinematics play a critical role in understanding gait mechanics, especially in overweight and obese children, where issues of stability and alignment are prevalent. Meta-analyses have highlighted increased pelvic motion in the transverse plane and greater hip internal rotation and flexion in obese individuals as characteristic features [15,16,52]. These findings are consistent with Horsak et al. [53], who noted asymmetry in pelvic motion and its impact on balance and motor control. Additionally, soft tissue artifacts, particularly around the pelvis and thighs, pose challenges in accurately assessing pelvic kinematics [53].

Despite these challenges, the current study aligns with broader evidence that overweight and obese children demonstrate greater pelvic and hip rotation in the transverse plane, contributing to instability during gait [15,16,54]. This increased motion has been associated with greater knee movement in the frontal plane and prolonged stance phases, as reported by Pathare et al. [50] and Cimolin et al. [51]. By introducing symmetry indices (SIT, SIO, SIR) across three planes of motion, the current study provided a detailed analysis of pelvic angular movement. Results showed lower symmetry values in the sagittal plane, consistent with earlier findings that overweight and obese children face challenges in maintaining pelvic balance during walking [13,51].

Unlike global metrics such as the harmonic ratio (HR), which quantifies step-to-step symmetry [51,55,56], the use of symmetry indices in this study allowed for a more specific evaluation of pelvic kinematics. These results offer insights into the direct impact of interventions on movement imbalances and stability.

The kinetic program applied in this study targeted flexibility, strength, and coordination, with the goal of improving both gait mechanics and balance. Programs with similar objectives, such as those examined by Horsak et al. [53], demonstrated significant improvements in walking speed and stride length after strength-based interventions. The researchers applied a complete 8-week lower extremity strength training program. Hip flexion moment and hip power generation represented pelvic kinematics studied parameters. By adopting the program and carrying it out daily, the child patient is able to efficiently engage the muscular chains of the lower limb with an efficient energy consumption within the walking pattern [8]. Nantel et al. [57] observed that obese children often rely on passive hip strategies to achieve forward progression, a less energy-efficient mechanism. The exercises in the current program successfully addressed these inefficient movement patterns by targeting major muscle groups and proprioceptive chains, leading to more balanced and coordinated gait mechanics. Compared with normal-weight children, overweight children had greater absolute peak joint moments at the hip (flexor, extensor, abductor, external rotator), the knee (flexor, extensor, abductor, adductor, internal rotator), and the ankle (plantar flexor, inverter, external/internal rotators). Overweight children experienced increased joint moments, which can have long-term orthopedic implications and suggest a need for more non-weight-bearing activities within exercise prescription. 

Meta-analyses further emphasize the importance of prolonged interventions. Steinberg et al. [58] demonstrated that longer-duration programs can significantly enhance gait parameters and reduce excessive forces at the foot level. Although the current program was limited to 60 min per session, the observed improvements suggest that even relatively short interventions can yield meaningful results. However, the absence of significant changes in certain parameters, such as stride length, highlights the need for longer or more intensive programs to achieve comprehensive improvements.

The findings from this study align with meta-analyses and individual studies that emphasize the unique gait adaptations in overweight and obese children, including increased step width, prolonged stance phase, and altered pelvic kinematics. The use of self-selected walking speeds in this study provided a realistic assessment of natural gait mechanics, complementing the controlled-speed conditions explored in other research [48,59]. These insights reinforce the need for tailored interventions that address both the spatio-temporal parameters and pelvic kinematics, with an emphasis on improving stability, balance, and energy efficiency during gait.

Future research should explore long-term outcomes of kinetic programs and incorporate a broader range of gait parameters, such as stance phase and step width, to provide a more comprehensive understanding of obesity-related gait adaptations. Additionally, advanced technologies like 3D motion capture systems and instrumented walkways could further refine the analysis of biomechanical dynamics. By addressing these gaps, future studies can build on the current findings to develop more effective rehabilitation strategies for improving the mobility, balance, and quality of life of overweight and obese children.

The present study offers several novel contributions to understanding and improving gait and movement in overweight and obese children, with practical implications for clinical and therapeutic applications:

- First Detailed Analysis of Pelvic Symmetry in Three Planes: By introducing symmetry indices (SIT, SIO, SIR) to evaluate pelvic motion in the sagittal, frontal, and transverse planes, this study provides a nuanced understanding of movement imbalances. Unlike traditional global measures such as the harmonic ratio, this method allows clinicians to pinpoint specific biomechanical deficits, enabling targeted interventions for correcting gait patterns and enhancing stability. Clinicians can use these symmetry indices to develop personalized rehabilitation programs focused on improving balance and coordination, reducing the risk of long-term musculoskeletal complications in overweight and obese children.
- Detailed Analysis of Pelvic Symmetry in Three Planes: By introducing symmetry indices (SIT, SIO, SIR) to evaluate pelvic motion in the sagittal, frontal, and transverse planes, this study provides a nuanced understanding of movement imbalances. Unlike traditional global measures such as the harmonic ratio, this method allows clinicians to pinpoint specific biomechanical deficits, enabling targeted interventions for correcting gait patterns and enhancing stability. Clinicians can use these symmetry indices to develop personalized rehabilitation programs focused on improving balance and coordination, reducing the risk of long-term musculoskeletal complications in overweight and obese children.
- Integration of a Comprehensive Kinetic Program: The kinetic program was uniquely designed to include flexibility, strength, and coordination exercises while addressing proprioception and balance. Special attention was given to restoring kinetic chains (triple flexion and triple extension) and strengthening key muscle groups such as the gluteus medius. These elements not only correct musculoskeletal deviations but also improve functional stability and reduce injury risk. This program can be easily adapted in rehabilitation and physiotherapy practices to improve gait mechanics, reduce the risk of falls, and enhance physical capabilities in overweight and obese children.
- Use of Accessible Gait Analysis Technology: The use of the BTS G-WALK/BTS G-SENSOR 2 device highlights the feasibility of conducting sophisticated gait analysis with portable and cost-effective tools. Compared to expensive and complex technologies like 3D motion capture or force platforms, this approach is more practical and scalable for routine clinical use. Health professionals, including physiotherapists and pediatricians, can incorporate this technology into their practice to monitor gait parameters and evaluate the progress of interventions in an efficient and affordable manner.
- Holistic Approach to Gait Analysis: By extending the analysis beyond the lower extremities to include the center of mass and pelvic kinematics, the study emphasizes the importance of the pelvis in maintaining balance and alignment during physical activity. This approach underscores the relationship between pelvic motion and overall gait stability. Rehabilitation programs can use this holistic perspective to address both localized and systemic movement issues, improving overall functional outcomes and reducing the risk of imbalance-related injuries.

However, this study is not without its limitations. First, the relatively short duration of follow-up: Although the study lasted six months, it may not have been sufficient to observe long-term effects of the kinetic program on gait mechanics and pelvic kinematics. Some parameters, such as stride length, may require more time for substantial changes to emerge. Second, self-selected walking speed: The use of self-selected walking speed provides a realistic assessment of natural gait but limits comparability with studies that use fixed-speed protocols. This could introduce variability in outcomes and make comparisons across studies less consistent. Third, the potential for measurement bias: the reliance on a single device for gait analysis, while accessible and cost-effective, may introduce limitations in precision compared to more sophisticated tools such as 3D motion capture systems or force platforms. Another limitation of this study is that it focused only on global spatio-temporal gait parameters, without examining the program’s impact on specific phases of the gait cycle (such as the stance phase, first double support phase, swing phase, and single support phase). This limits the ability to fully understand how the kinetic program affects the entire gait cycle. Additionally, the study did not include direct measurements of balance, coordination, and strength, which are hypothesized to play a significant role in the observed outcomes. The absence of such measurements limits our ability to conclusively link these factors to the improvements noted in the Study Group. Also, the potential Hawthorne effect, where participants in the SG may have altered their behavior due to being observed, could influence outcomes. Furthermore, reliance on self-reported adherence to educational measures and activity recommendations introduces the possibility of reporting bias.

Addressing these limitations in future research, such as including diverse populations, assessing additional gait parameters, and conducting long-term follow-ups, could enhance both the robustness and applicability of the results.

This study demonstrates notable strengths in internal validity. The comparability of baseline characteristics between the SG and CG, with no significant differences in key parameters, ensured a robust starting point for assessing the intervention’s effects. Natural growth and development over the six-month period were accounted for, reducing confounding variables unrelated to the intervention. Additionally, the use of standardized protocols and a single device (BTS G-WALK) minimized variability in data collection, reinforcing the reliability of the results.

In terms of external validity, the study’s use of portable and accessible technology enhances its reproducibility in clinical and community settings. The focus on clinically relevant gait parameters, such as cadence, speed, and stride length, increases the applicability of the findings to broader healthcare contexts.

## 5. Conclusions

The findings of this study are highly relevant for clinical practice, particularly in the rehabilitation of overweight and obese children. The use of affordable, portable technology and clinically relevant metrics allows for the integration of evidence-based gait analysis and intervention programs into routine care. The kinetic program’s emphasis on flexibility, strength, and coordination provides a replicable model for improving gait stability and biomechanical efficiency in this population.

By addressing gait abnormalities and balance deficits, the proposed interventions can improve the quality of life for overweight and obese children, reducing the long-term risk of musculoskeletal issues and promoting lifelong physical activity. Future research should focus on refining these interventions, exploring their long-term impact, and expanding their applicability to broader populations.

## Figures and Tables

**Table 1:** Kinetic program indicated to the children (SG).

Kinetic Objective	Description
Warm-Up/10 min Gentle walking followed by light stretching focusing on the calves, thighs, hips, and lower back.	
Balance Exercises	10 min Standing on one leg, alternating every 30 s. Walking heel-to-toe in a straight line to simulate balance beam exercises.
Coordination Exercises	10 min Walking forwards and backwards with quick directional changes on cue. Simple ball games, such as throwing and catching balls of different sizes.
Strength training	20 min Squats: 3 sets of 8–10 repetitions to strengthen the thighs and buttocks. Wall pushes: 3 sets of 10 repetitions to build upper body and core strength. Calf raises: 3 sets of 15 repetitions to strengthen the lower legs. Hip Bridges: 3 sets of 10 repetitions. This exercise is excellent for strengthening the hip muscles and stabilizing the lower back. Side leg raises: 3 sets of 10 repetitions on each side to target the hip abductors, crucial for stable walking.
Aerobic Activities	20 min Bike ride/brisk walking or light jogging These aerobic activities restore/maintain the ability to perform previous exercises and have a beneficial impact on the bone structure.
Cool Down—5 min Gentle walking and light stretching to relax the muscles and prevent stiffness.	

The strength training was performed using body weight, without using traditional resistance.

**Table 2:** Anthropometric data at the T1 moment.

	SG 45 Children	CG 48 Children	*p* Value
Age (years)	10.39 ± 1.97	10.96 ± 2.15	0.1145
Boys (n,%)	22 (49%)	23 (48%)	0.2621
Girls (n,%)	23 (51%)	25 (52%)	0.2172
Urban (n,%)	23 (51%)	27 (56%)	0.2624
Rural (n,%)	22 (49%)	21 (44%)	0.3124
Weight (kg)	52.89 ± 15.84	58.12 ± 15.68	0.2980
Height (cm)	134.10 ± 17.65	139.56 ± 14.54	0.3160
BMI (Kg/m^2^)	29.49 ± 4.06	29.40 ± 3.43	0.3536

Variables are reported as mean ± (SD) (standard deviation), n = number of subjects, % = percent of subjects, BMI = Body Mass Index, and *p* = *t*-test for independent samples.

**Table 3:** Mean values of evaluated parameters in both assessment moments (initial/final).

	SG Mean	SD	CG Mean	SD	*p*-Values
BMI_1 (kg/cm^2^)	29.49721	4.066419	29.40969	3.439942	0.727
BMI_2 (kg/cm^2^)	28.85294	3.279355	29.66154	2.916960	0.077
cadence1 (step/min)	118.7941	14.04849	116.2308	10.83941	0.135
cadence2 (step/min)	121.4559	13.93314	116.8615	10.68129	**0.015**
speed1 (m/s)	1.261471	0.461698	1.172308	0.352348	0.341
speed2 (m/s)	1.439118	0.449035	1.195077	0.336197	0.0066
stride length1 (meter)	1.200000	0.318626	1.211231	0.358981	0.790
stride length2 (meter)	1.3315588	0.333495	1.238308	0.346304	0.071
SIT1	57.01471	22.95778	52.43077	10.68581	0.220
SIT2	71.44118	18.53903	52.80000	10.40162	**<0.001**
SIO1	93.55882	5.415242	94.13846	3.673711	0.685
SIO2	97.83824	1.288413	93.95385	3.420667	**<0.001**
SIR1	92.22059	11.16076	93.32308	5.282955	0.484
SIR2	96.02941	7.532954	94.36923	4.453121	**<0.001**

SIT1—symmetry index for pelvic tilt motion (T1); SIT2—symmetry index for pelvic tilt motion (T2); SIO1—symmetry index for pelvic obliquity motion (T1); SIO2—symmetry index for pelvic obliquity motion (T2); SIR1—symmetry index for pelvic rotation motion (T1); SIR2—symmetry index for pelvic rotation motion (T2); BMI1 = body mass index (T1); BMI2 = body mass index (T2); cadence1—cadence (T1); cadence2—cadence (T2); speed1—speed (T1); stride length1—stride length (T1); stride length1—stride length (T1); *p*-values were established with the Mann–Whitney U test (the significant values are in bold).

## Data Availability

Data are contained within the article.
